# Electronic Localization Derived Excellent Stability of Li Metal Anode with Ultrathin Alloy

**DOI:** 10.1002/advs.202105656

**Published:** 2022-02-04

**Authors:** Danqi He, Wenjun Cui, Xiaobin Liao, Xianfei Xie, Mingheng Mao, Xiahan Sang, Pengcheng Zhai, Yan Zhao, Yunhui Huang, Wenyu Zhao

**Affiliations:** ^1^ Hubei Key Laboratory of Theory and Application of Advanced Materials Mechanics Wuhan University of Technology Wuhan 430070 China; ^2^ State Key Laboratory of Advanced Technology for Materials Synthesis and Processing Wuhan University of Technology Wuhan 430070 China; ^3^ State Key Laboratory of Advanced Electromagnetic Engineering and Technology School of Electrical and Electronic Engineering Huazhong University of Science and Technology Wuhan 430074 China; ^4^ State Key Laboratory of Material Processing and Die and Mold Technology School of Materials Science and Engineering Huazhong University of Science and Technology Wuhan 430074 China

**Keywords:** electronic localization, high capacity, Li metal anode, self‐smoothing effect, ultrathin alloy

## Abstract

Lithium metal is an ideal anode for next‐generation high‐energy‐density batteries. However, lithium dendrite growth has impeded its commercial application. Herein, fabricating Li‐based ultrathin alloys with electronic localization and high surface work function via depositing Bi, Al, or Au metals on the surface of copper foil for in situ alloying with lithium is proposed. It is discovered that the electronic localization can induce self‐smoothing effect of Li ions, as a result, significantly suppressing the growth of dendritic lithium. Meanwhile, the high surface work function can effectively alleviate side reactions between the electrolyte and lithium. With the as‐obtained ultrathin alloys as anodes, excellent cycling performance is achieved. The half cells run stably after more than 120 cycles under high capacity of 4 mAh cm^−2^. The S||Bi/Cu‐Li full cell delivers a specific capacity of 736 mAh g^−1^ after 200 cycles. This work provides a new strategy for fabricating long‐life and high‐capacity lithium batteries.

## Graphical abstract

The strong localized electrons on the surface of Li‐based ultrathin alloys can attract Li nuclei, and help dissolve Li clusters into isolated Li nuclei to produce self‐smoothing effect of Li ions, realizing the flat and dense deposition of Li and significantly suppress the growth of dendritic lithium during the entire plating process.

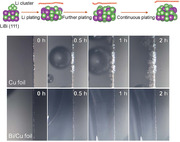

## 1 Introduction

Energy storage devices with high energy density, good safety, and excellent stability are necessary and urgent for the development of next‐generation battery technologies.^[^ ^1^ ^]^ The electrode materials are critical to determine the energy density.^[^ ^2^ ^]^ Lithium metal is considered as the ultimate anode material due to exceptional theoretical specific capacity of 3860 mAh g^–1^ and extremely low redox potential of −3.04 V versus standard hydrogen electrode.^[^ ^3^, ^4^ ^]^ The theoretical energy density even reaches 2567 (3505) Wh kg^–1^ by pairing with sulfur (air) cathode.^[^ ^5^, ^6^ ^]^ However, a major problem associated with Li metal anode is the formation of Li dendrites because of the non‐uniform deposition of Li^+^ during cycling.^[^ ^7^ ^]^ The Li dendrites can easily detach from the electrode to form “dead Li,” which would accumulate on the surface of the electrode to accelerate the capacity decay and greatly increase the internal resistance of batteries. This eventually leads to reducing Coulombic efficiency (CE) and shortening cycle life. Moreover, the Li dendrites may still pierce through the separator and cause short circuit, resulting in safety issues such as fire and explosion, which severely limits the practical application of Li metal anode.^[^ ^8^, ^9^, ^10^ ^]^

To date, many efforts have been devoted to protecting Li metal anode. One important strategy is to alleviate the high reactivity of Li through various approaches, such as employing stable electrolyte to avoid side reactions,^[^ ^11^, ^12^ ^]^ or fabricating solid electrolyte interphase layer to prevent the side reaction between Li metal and electrolyte.^[^ ^13^, ^14^, ^15^, ^16^ ^]^ Another effective strategy is to promote the uniform deposition of Li^+^ ions by inducing the Li^+^ electroplating using active sites or nanostructures,^[^ ^17^, ^18^, ^19^ ^]^ reducing the heterogeneous nuclear barrier utilizing lithiophilic groups,^[^ ^20^, ^21^ ^]^ or decreasing current density via 3D current collectors with high specific surface area.^[^ ^22^, ^23^, ^24^ ^]^ Unfortunately, up to now, fabricating stable Li metal anode beyond 3 mAh cm^−2^ is still an unfulfilled goal with extreme difficulty. The main reason is that all methods mentioned above only focus on the nucleation of Li or the initial period of Li growth, thus they cannot ensure a large amount of subsequent Li^+^ to deposit uniformly due to the tip effect. Therefore, achieving a sustainably homogeneous distribution of Li ions during the entire plating process is of critical importance toward ultralong cycle life for the battery with high areal capacity.

In this work, we propose to fabricate Li‐based ultrathin alloys with electronic localization and high surface work function (SWF) via depositing Bi, Al, or Au metals on the surface of copper foil for in situ alloying with lithium. We discover that the localized electrons on the surface of Li‐based ultrathin alloys strongly attract Li nuclei, and help dissolve Li clusters into isolated Li nuclei. This self‐smoothing effect can completely suppress the formation of Li dendrites on the alloy surface for subsequent deposition. Moreover, the high SWF of Li‐based ultrathin alloys can slow down electron transfer from Li atoms, which effectively alleviates side reactions between Li and electrolyte. Using these ultrathin Li‐based alloys as anodes, the electrochemical performance is greatly improved. This work provides a new strategy for fabricating long‐life and high‐capacity lithium batteries.

## 2 Results and Discussion

**Figure 1:**
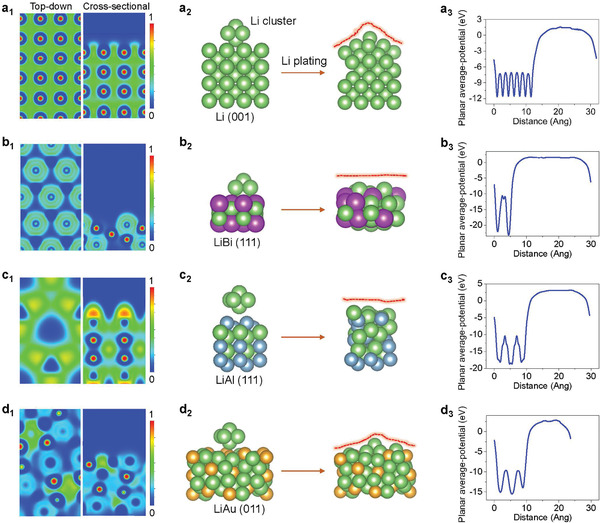
Theoretical prediction of self‐smoothing behaviors for electrochemical Li deposition on lithium alloy interphases. ELF results of the a_1_) Li (001), b_1_) LiBi (111), c_1_) LiAl (111), and d_1_) LiAu (011). The deposition of Li cluster on the surface of a_2_) Li, b_2_) LiBi, c_2_) LiAl, and d_2_) LiAu. SWF of a_3_) Li, b_3_) LiBi, c_3_) LiAl, and d_3_) LiAu.

The electron localization function (ELF) has been simulated as the useful metric for characterizing the free electron distribution.^[^ ^25^, ^26^ ^]^ ELF with large amount of localized free electrons also implies the electron transfer pathway and the potential deposition sites for ions.^[^ ^27^, ^28^ ^]^ To explore the effect of electronic localization from lithium alloy on the depositing behaviors of Li^+^, we selected three Li‐based alloys (LiBi‐cubic alpha bismuth trifluoride structure with Fm$\bar{3}$m space group, LiAl‐cubic NaTl structure with Fd$\bar{3}$m space group, and LiAu‐cubic crystal structure with I$\bar{4}$3d space group) with different degrees of electronic localization and performed the surface electronic property and Li cluster deposition calculations. The ELF results of the Li(001), LiBi(111), LiAl(111), and LiAu(011) surfaces are shown in **Figure** **1**a_1_–d_1_, respectively. Free electrons are uniformly distributed on the surface and in the bulk phase of lithium metal (Figure 1a_1_), which leads to the same probability for the deposition of Li^+^ ions on the surface. When depositing a Li cluster on the Li(001) surface, after a full relaxation, the Li cluster is absorbed on the Li metal surface and the structure of the cluster remains (Figure 1a_2_), which leads to a tip shape of Li surface. As shown in ELF result (Figure S1, Supporting Information), the undecomposed cluster shows strong localized distribution. This reveals that the uniform electron distribution shows less function on regulating the Li deposition and decomposition of Li cluster. By a sharp comparison, the lithium alloys (LiBi, LiAl, LiAu) show localized distribution of free electrons on the surface (Figure 1b_1_–d_1_). Specifically, the free electrons are concentrated around the Bi or Al atoms with the value of 0.5 and 0.7, respectively, and these sites with localized electrons may show the affinity to Li^+^ and regulate the deposition behavior via the efficient electron transfer process. Whereas at the LiAu surface, the free electrons are concentrated around the Li atoms and the value of ELF around the Au atoms is as low as 0.2, implying the weak effect on regulating Li^+^. As expected, the Li clusters on both LiBi and LiAl surfaces are completely decomposed (Figure 1b_2_,c_2_ and Figures S2 and S3, Supporting Information), and that on the LiAu surface is decomposed to isolated Li_5_ cluster and one Li atom (Figure 1d_2_ and Figure S4, Supporting Information). This result suggests that the deposited Li on the LiBi and LiAl surfaces can realize “self‐smoothing effect.” Moreover, after platting a Li cluster, the localized electrons in LiBi are still evenly distributed around Bi atoms (Figure S2, Supporting Information), but the electrons seem to form unfavorable segregation in LiAl (Figure S3, Supporting Information), demonstrating that the LiBi alloy is more advantageous in the adsorption and decomposition of Li cluster.

The deposition process of Li ions is also affected by the reaction of electrolytes. The ability for suppressing the parasitic reaction of the electrolytes is evaluated by the SWF, which represents the minimum energy needed to remove an electron from a solid to a point immediately outside the solid surface.^[^ ^29^ ^]^ The calculated SWF of the pure Li metal is only 2.9 eV (Figure 1a_3_: Table S1, Supporting Information), indicating the free electrons on Li surface are easily transferred to the electron‐absorbing groups (such as nitrate, ether bond and amine group) in the electrolyte during the reaction, thus resulting in a series of reductive decomposition reactions. In contrast, the artificial LiBi, LiAl, and LiAu alloys show relatively higher SWF values of 4.4, 4.1, and 3.6 eV, respectively, implying that the activity of the Li metal is effectively reduced after alloying reaction. Therefore, it is theoretically predicted that lithium alloys can effectively inhibit lithium dendrites and protect the Li metal anode through the intrinsic localized electronic and high SWF.

**Figure 2:**
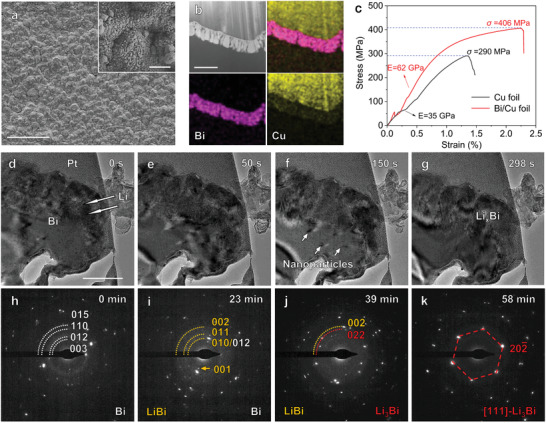
Design and fabrication of ultrathin lithium alloy layer. a) The surface morphology of Bi/Cu foil, and the inset is the corresponding enlarge pictures. Scale bars, a) 20 µm and inset 1 µm. b) HAADF image of the Bi/Cu foil and the corresponding EELS mappings of Cu and Bi elements for Bi/Cu foil. Scale bars, 500 nm. c) Typical tensile stress‐strain curves of the Cu foil and Bi/Cu foil. d–g) Evolution of morphology and microstructure of Bi/Cu foil, when a bias of 0.1 V is applied, a lithiation process is triggered. Scale bars, 200 nm. h–k) The dynamic electron diffraction pattern evolution of Bi/Cu foil during lithiation reaction, and a bias of 1 V is applied.

To simply introduce lithium alloy into the lithium anode, ultrathin metal film was deposited on the surface of the commercial Cu foil, and then reacted with Li to in situ form an ultrathin alloy layer during electrochemical process. Compared with the common slurry‐coating and vacuum filtration methods, the magnetron sputtering can realize large‐scale production of uniform thin film with good mechanical stability, and thus was used to deposit Bi film onto the Cu foil to obtain Bi/Cu foil. After magnetron sputtering, the rough and ribbed surface of the Cu foil (Figure S5, Supporting Information) is covered by a layer of evenly distributed fine particles (**Figure** **2**a). From the X‐ray diffraction (XRD) patterns (Figure S6, Supporting Information), it can be confirmed that the fine particles in the Bi/Cu foil are pure bismuth without bismuth oxides. This is attributed to the high vacuum of magnetron sputtering and the strong antioxidant activity of bismuth. This also implies that the current collector is not easily oxidized if sputtering Bi on the Cu foil during the production process, which is beneficial for the conductivity of the battery. From the high angle annular dark‐field (HAADF) image and the corresponding elemental mappings, it can be found that the uniform and dense Bi film with a thickness of about 300 nm tightly adheres to the surface of the Cu foil (Figure 2b). Although the modified layer is very thin, the small particle size of bismuth and its tight adhesion to the rough surface of Cu foil can enhance the mechanical deformation property of Cu foil, as shown in the stress–strain curves (Figure 2c). The ultimate strength and elastic moduli of Bi/Cu foil are 406 MPa and 62 GPa, respectively, larger than those of Cu foil, demonstrating that the Bi/Cu current collector is more suitable for industrial transport and applications.

*In‐situ* transmission electron microscopy (TEM) imaging was employed to investigate the formation process of lithium alloy (Figure 2d‐g: Movie S1, Supporting Information). When Li ions start to deposit on the Bi/Cu foil along the direction marked by the white arrows in Figure 2d, the Li cluster shows continuous volume shrinkage and Bi layer undergoes deformation such as buckling (Figure 2e–g), implying that Li has been incorporated into the Bi film and participated in in situ reaction to form nanoparticles. The in situ selected area electron diffraction (SAED) (Figure 2h–k: Movie S2, Supporting Information) confirms that Bi thin film, consisting of pure Bi with $R\bar{3}$m crystal structure (Figure 2h), first reacts with Li to form intermetallic compound LiBi with tetragonal crystal structure (space group P4/mmm) (Figure 2i), where diffraction rings from LiBi (001), (010) and (001) planes are marked by orange arcs. And then LiBi and Li continue to form Li_3_Bi with cubic crystal structure (space group Fm$\bar{3}$m), and the corresponding diffraction spots are delineated by red arcs (Figure 2j). After adequate reaction, the original Bi film on the Bi/Cu foil has completely transferred into a single grain of Li_3_Bi oriented along [111] zone axis (Figure 2k), of which the theoretical specific capacity is 384.8 mAh g^−1^ (based on the mass of Bi). Combining experimental results (Figures S7 and S8, Supporting Information), we can conclude that the metal deposited on the Cu foil can be successfully transferred into lithium alloy, and the in situ formation process can be described by two separate steps:

(1) $$\mathrm{First}\mathrm{lithiation}:\mathrm{Bi}+Li^{+}+e^{-}\to \mathrm{LiBi}$$

(2) $$\mathrm{Second}\mathrm{lithiation}:\mathrm{LiBi}+2Li^{+}+2e^{-}\to Li_{3}\mathrm{Bi}$$

**Figure 3:**
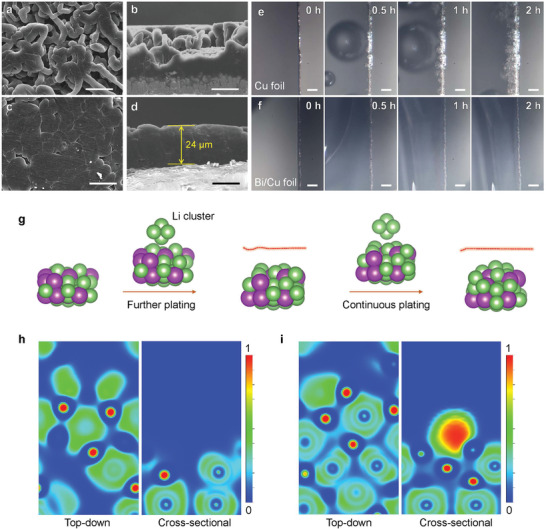
Li growth regulated by the electronic localization. SEM images of Li deposition on a,b) Cu foil and c,d) Bi/Cu foil at a current density of 1 mA cm^–2^ with a total capacity of 4 mAh cm^–2^ (scale bar: 20 µm). *In‐situ* optical microscopy of Li deposition process on e) Cu foil and f) Bi/Cu foil at 1 mA cm^−2^ (scale bar: 50 µm). g) The continuous deposition of Li cluster on the surface of LiBi. ELF results of LiBi after h) two Li clusters and i) three Li clusters platting.

Based on the determination of the above characterization results, we used the Bi/Cu foil to verify the effect of electronic localization on the Li deposition behavior by SEM and in situ optical microscopy. With Cu foil as current collector, the deposited Li has many cracks and rough surface (Figure S9a, Supporting Information); increasing Li content brings a lot of dendritic Li, porous and dead Li, which is resulted from inhomogeneous Li deposition (**Figure** **3**a,b: Figure S9b, Supporting Information). In sharp contrast, the surface morphology of Li deposited on Bi/Cu current collector is much flatter and denser (Figure S9c,d, Supporting Information). Even after plating a capacity of 4 mAh cm^–2^, the surface is still very smooth and no porous or dendritic Li appears (Figure 3c). From the cross‐sectional image (Figure 3d), we can see that, after plating 4 mAh cm^−2^, the thickness of the deposited lithium on the Bi/Cu foil is about 24 µm, which approaches that on Li foil with a content of 4 mA h cm^−2^ (20 µm), further indicating that the Li_3_Bi layer formed in the initial cycles can effectively induce Li depositing and lead to extremely dense deposited lithium with no dead lithium or dendrite. Therefore, the Bi/Cu current collector enables longer lifespan of a cell, even at a high total capacity. In situ optical microscopy was further applied for characterizing the deposition process of Li and confirm the stability of Bi/Cu current collector. For the bare Cu foil, the deposited metallic Li grains with gray color appear on the surface with prolonging plating time, the surface of bare Cu foil is gradually covered by needle‐like Li dendrites, revealing inhomogeneous distribution of Li^+^ and disordered growth of Li metal on commercial Cu foil (Figure 3e). In comparison, Bi/Cu electrode exhibits superior protection on metallic Li. Although the plating time lasted for 2 h, there is no appearance of gray Li dendrite on the surface of Bi/Cu foil as compared to its initial state (Figure 3f). These results demonstrate that, in the experiment, the self‐smoothing behavior of Li at the lithium alloy layer is extremely effective to promote uniform and dense deposition of Li^+^ ions. Computationally, when more Li clusters are deposited on the Li_3_Bi phase (Figure 3g), the Li clusters are still continuously decomposed and show self‐smoothing behavior. From the corresponding ELF, with the second Li cluster platting, the localized electrons are still concentrated around Bi atom to regulate the deposition behavior via the efficient electron transfer process (Figure 3h). By continuously platting Li cluster (Figure 3i), from the top‐down view, the localized electrons of the surface are widely distributed and the value of ELF around the Bi atoms is a little lower. But from the cross‐sectional view, we are surprised to find an area with very strong localized electrons to adsorb lithium atoms, and the area is the void formed by four Li atoms, which can explain why the metal Li deposited on Bi/Cu foil is very flat and dense. This deep calculation powerfully matches the experimental results of uniform Li deposition with different capacities (Figure 3c,d,f). Moreover, this implies that the lithium alloy layer can not only induce the lithium deposition in the initial stage, but also achieve a sustainably homogeneous distribution of lithium ions during the entire plating process.

**Figure 4:**
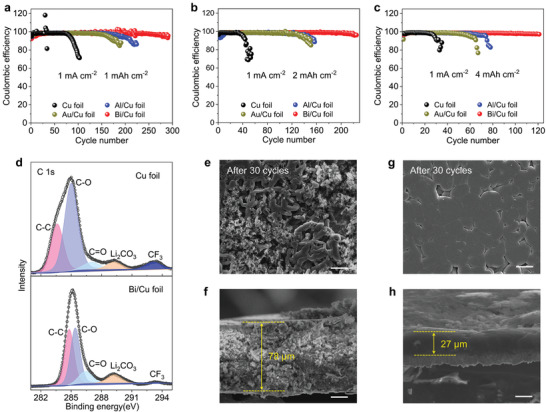
Electrochemical data of half‐cells. a–c) CE of different current collectors at current density of 1 mA cm^–2^ with a total capacity of a) 1 mAh cm^–2^, b) 2 mAh cm^–2^, and c) 4 mAh cm^–2^. d) XPS spectra of C 1s for different current collectors after cycling. e–h) SEM images of the morphologies of Li deposition with a capacity of 4 mAh cm^–2^ on e,f) Cu and g,h) Bi/Cu current collectors after 30 depositing/stripping cycles (scale bar: 20 µm).

Based on the above prediction of the effect of electronic localization from lithium alloy layer on the uniform Li^+^ deposition, and because the CE is essential for maintaining a long cycle life of battery, we further studied the efficiency of Li deposition through asymmetric half cells. **Figure** **4**a–c compares the CE of different electrodes (Cu, Bi/Cu, Al/Cu, and Au/Cu) at a fixed current density of 1 mA cm^−2^ with various capacities. Obviously, all the current collectors with lithium alloy layer show much longer cycle life‐span under all different capacities. Specifically, with Li content of 1 mAh cm^–2^, the CE of Cu foil drops to 70% after only 100 cycles. In contrast, with the introduction of lithium alloy layer, the CE of the half cells Li||Bi/Cu, Li||Al/Cu, and Li||Au/Cu remain much more stable. Especially, the Li||Bi/Cu cell could steadily run for over 300 cycles with high CE values due to the self‐smoothing effect induced by localized electrons. With increasing the areal capacity to 2 mA h cm^−2^, the Cu foil starts to drop after only 30 cycles and decreases to about 70% over 40 cycles. The Al/Cu and Au/Cu foil can steadily run for about 120 cycles. Moreover, the CE of the Li metal half cell with Li_3_Bi layer keeps at about 99.1% after 200 plating/stripping cycles. Even cycling under a high Li content of 4 mAh cm^–2^, the Bi/Cu electrode is still stable for more than 120 plating/stripping cycles (Figure 4c), indicative of good reversibility and high utilization of Li during Li plating and stripping, and the corresponding voltage profiles of Bi/Cu electrode show no change at different cycles (Figure S10, Supporting Information). From the above analysis, we conclude that the three kinds of lithium alloy layers all show protection effect on Li metal anode due to the self‐smoothing behavior of Li on lithium alloy layer, especially under a high Li content. Since the LiBi alloy can smooth Li deposition in the entire plating process, Li||Bi/Cu cell exhibits excellent performance. Additionally, although the half cells using the Au/Cu and Al/Cu foil display longer cycling life than the bare Cu foil, they are inferior to the Bi/Cu foil, which is attributed to the weak electronic localization of LiAu alloy and the uneven distribution of electronic localization of the LiAl alloy. Meanwhile, lithium alloys with high SWF have poor electron transfer ability, which can reduce the reaction activity between the Li and electrolyte. To confirm this point, we studied surface chemical composition of the bare Cu and Bi/Cu electrodes after five depositing/stripping cycles using X‐ray photoelectron spectroscopy (XPS) (Figure 4d: Figure S11, Supporting Information). In C 1s spectra, the surface of Cu foil shows obvious peak for CF_3_ and strong peak for C‐O, indicating the decomposition of electrolyte. However, in the surface of Bi/Cu foil, because the SWF of Li_3_Bi is up to 4.4 (Figure 1b_3_: Table S1, Supporting Information), there is almost no CF_3_ peak appearing and the peak for C‐O is relatively weak, suggesting that the Bi/Cu foil could mitigate side reactions between electrolyte and Li anode and contribute to the improved Li utilization and stability. From the FESEM image of deposited Li after 30 plating/stripping cycles (1 and 4 mAh cm^–2^), we can see that the deposited Li on bare Cu foil is occupied by Li dendrite and dead Li (Figure 4e) with thickness up to 78 µm (Figure 4f), far exceeding that of lithium foil with 4 mAh cm^–2^. This explains why the CE drops seriously and cannot proceed to work. By contrast, due to the self‐smoothing behavior of Li on the alloy layer and its high SWF, the deposited Li on Bi/Cu foil is still smooth and dense (Figure 4g,h) with thickness of only 25 µm, approaching that on Li foil, which also provides the evidence for good long‐cycling stability of Bi/Cu foil.

**Figure 5:**
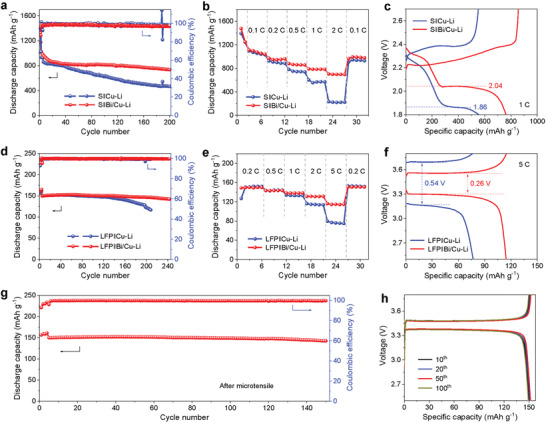
Electrochemical characterizations of full cells. a) Cycling performance at 0.2 C, b) rate performance and c) the corresponding voltage profiles of the full‐cells consisting of a Cu–Li or Bi/Cu–Li anode and a sulfur cathode. d) Cycling performance at 1 C, e) rate performance and f) the corresponding voltage profiles of the full‐cells consisting of a Cu‐Li or Bi/Cu‐Li anode and a LFP cathode. g) Cycling stability at 0.5 C of LFP||Bi/Cu‐Li full‐cell using Bi/Cu foil after microtensile and h) the corresponding discharge/charge curves at the 10th, 20th, 50th, and 100th cycle.

Full cells based on S or LiFePO_4_ (LFP) cathodes were fabricated to investigate the electrochemical performance of Bi/Cu current collector under practical conditions. As expected, Bi/Cu current collector demonstrates better cycling performance than the bare Cu foil. Particularly, because localized electrons can effectively protect Li anode, the S||Bi/Cu‐Li cell delivers a specific capacity of 736 mAh g^−1^ at a fixed current of 0.2 C with stable CEs, far higher than 466 mAh g^−1^ of the S||Cu‐Li cell (**Figure** **5**a). Moreover, since the lithium alloy layer can reduce the internal resistance, as well as suppress side reactions due to the high SWF under high current condition, enhanced rate performance is attained with reversible capacities of 972, 881, 790, and 698 mAh g^−1^ at 0.2, 0.5, 1, and 2 C, respectively, superior to the performance of the bare Cu–Li cell with a specific capacity of 220 mAh g^−1^ at 2 C (Figure 5b). Figure 5c compares the charge/discharge curves at 1 C between S||Bi/Cu–Li and S||Cu–Li cells. The former shows higher typical plateaus than the latter, which is attributed to the faster ion transport kinetics from the lithium alloy phase. When paired with LFP cathode, LFP||Bi/Cu–Li exhibits stable cycling at 1 C over 240 cycles with an average capacity decay of 0.019% per cycle and stable rate capacity of 143, 138, 131, and 115 mAh g^−1^ at 0.2, 0.5, 1, 2, and 5 C, respectively (Figure 5d–f), which is also due to the self‐smoothing effect induced by ultrathin alloy with strong and uniform electronic localization contributing to the anode. On the contrary, bare Cu‐Li cell suffers rapid capacity decay after 120 cycles at 1 C and shows a much larger polarization at 5 C (0.54 V) (Figure 5f). Even when paired with high‐loading LFP cathode (11 mg cm^−2^), the LFP||Bi/Cu‐Li still shows better cycle stability at 0.2 C than the cell with bare Cu foil (Figure S12, Supporting Information). These results evidently support that lithium alloy with local electrons can effectively realize dendrite‐free lithium deposition to remarkably enhance capacity, cycle life, and rate performance of full cells.

To observe the influence of mechanical deformation of Bi/Cu current collector on the electrochemical performance, the full cell using the Li‐plated Bi/Cu foil after microtensile as the anode coupled with LFP cathode was cycled at 0.5 C, which delivers the unaffected performance of 150 mAh g^−1^ with a stable CE of 99.7% and capacity retention of 142 mAh g^−1^ after 150 cycles (Figure 5g). From the charge–discharge voltage profiles (Figure 5h), we can see that the LFP||Bi/Cu‐Li cell after microtensile exhibits very steady voltage profiles at different cycles, further demonstrating that the Bi/Cu foil due to the strong localized electrons of LiBi alloy can still effectively ensure the uniform deposition and stripping of Li through the unavoidable slight mechanical deformation.

## 3 Conclusion

In conclusion, we develop an efficient strategy by modifying the commercial copper foil with in situ formed ultrathin lithium‐metal alloy layer to achieve stable dendrite‐free Li metal anode, and first propose the mechanism that the localized distributions of free electrons on the surface of Li alloy, concentrated around the Bi or Al atoms, favor the affinity to the Li ions and the decomposition of Li cluster, which produce “self‐smoothing effect” during the initial state of Li platting. With continuously depositing Li clusters, the voids formed by the previously accumulated Li atoms with quite strong localized electrons can adsorb lithium atoms to realize the flat and dense deposition of Li. Meanwhile, the high SWF of lithium alloy can effectively alleviate side reactions between Li and electrolyte. Thus, the Bi/Cu foil exhibits stable cycling performance even under a high capacity of 4 mA h cm^−2^; the half cell can keep steady for more than 120 plating/stripping cycles. Being paired with S or LFP cathode, the Bi/Cu–Li exhibits obviously improved electrochemical performance compared to the counterparts without the lithium alloy layer. More importantly, we demonstrate that this self‐smoothing mechanism induced by electronic localization shed new light on the design principles of metallic Li anode and open up new opportunities to protect other alkali‐metal anodes such as Na and K.

## 4 Experimental Section

### Fabrication and Characterizations of the Modified Current Collector

The Bi/Cu, Al/Cu, and Au/Cu current collectors were fabricated by magnetron sputtering method. The modification layers were deposited on commercial copper foil under Ar atmosphere by using a magnetron sputtering system (JSD450, Anhui Jiashuo Vacuum Tech. Co., Ltd.).

### Materials Characterization

The phase constituents were determined by XRD (Bruker D8‐Advance) using a Cu *Kα* radiation. The morphologies of all electrodes were observed by field emission scanning electron microscopy (FEI Quanta 650 FEG). HAADF image and elemental mappings were performed on FEI Talos F200S microscope equipped with Super‐X EDX system, operated at 200 kV. In situ  TEM was performed by a TEM‐STM in situ sample holder (Zep Tools Co. Ltd., China) on a FEI Talos F200S TEM. Microtensile tests were carried out under displacement control by an Instron 5848 Microtester. Elastic moduli was determined in the linear elastic region of the stress–strain curves.

### Electrochemical Measurements

All the electrochemical properties were measured with standard CR2032 coin‐type cells, all which were assembled in the argon‐filled glove box under the conditions of the oxygen and water contents both below 0.1 ppm. The electrolyte used in the cells was 1 mol L^–1^ LiTFSI in a mixture of 1,3‐dioxolane (DOL) and dimethoxymethane (DME) (v/v = 1 : 1) with 2 wt.% of LiNO_3_. CR2032 coin cells were assembled with the bare Cu foil, Bi/Cu foil, Al/Cu foil or Au/Cu foil as the working electrode, the prepared ether electrolyte, separator of Celgard 2400 and a Li foil as the counter electrode. The cycling performance was measured on a Land Battery Measurement System (Land, China). To test the CE, the cells were firstly cycled at 0–1 V at 0.5 mA for three cycles. And then, different contents of Li (1, 2, and 4 mAh cm^–2^) were deposited on the working electrodes at a fixed current density of 1 mA cm^–2^ and then stripping away until the voltage reached up to 0.5 V. For full‐cell test, the sulfur cathode was made by mixing 80 wt.% S/Ketjen Black composites (S/Ketjen Black composites were sealed in a container at 155 °C for 12 h, by simply heating the mixture of sublimed sulfur and Ketjen Black carbon at accurate weight ratios of 7:3), 10 wt.% Super P, 10 wt.% LA133 to form a homogeneous slurry. The slurry was spread onto carbon paper and dried in air at 60 °C for 24 h, which resulted in the formation of S cathode with a sulfur loading of 3.0 mg cm^–2^. And the LiFePO_4_ cathode electrode was made by mixing 93 wt.% of LiFePO_4_ powders, 2.5 wt.% of conductive carbon (Super P and Ketjen Black with a mass ratio of 4:6), and 4.5 wt.% of PVDF to form a homogeneous slurry. The slurry was spread onto Al foil and dried in vacuum at 120 ℃ for 24 h. The mass loading of LiFePO_4_ in the electrode was ≈5 mg cm^–2^.

### Computation

Spin polarized density functional theory (DFT) simulations were conducted via Vienna Ab initio Simulation Package (VASP). The generalized gradient approximation with Perdew–Burke–Ernzerhof functional was selected for describing the exchange‐correlation of electrons. The interaction between core electrons and valence electrons was performed by the projector augmented wave. The DFT‐D3 method with Becke‐Jonson damping was added for the van der Waals. The cut off energy was set to 520 eV and the Brillouin zone was sampled by the gamma centered Monkhorst‐Pack Grid with k‐points of 1 × 1 × 1 for the structural optimization. The geometry optimizations were relaxed until the energy and residual force smaller than 10–5 eV and 0.02 eV/Å, respectively. The calculations of the ELF and SWF were performed by using Monkhorst‐Pack Grid with the k‐points of a*k_1_≈b*k_2_≈90, c*k_3_≈30 (a, b, and c were the lattice parameters of the structures). The DFT calculations of DOL, DME, LiNO_3_, LiTFSI were carried out using Gaussian 16 software package. The structures were fully optimized by using M062x level of theory with 6–311+g(2df,2p) basis set.

## Conflict of Interest

The authors declare no conflict of interest.

## Supporting information

Supporting Information

Supplemental Movie 1

Supplemental Movie 2

## Data Availability

Research data are not shared.
