# Supplementary material for: Electronic Localization Derived Excellent Stability of Li Metal Anode with Ultrathin Alloy
Source: Adv Sci (Weinh). 2022 Feb 4;9(10):2105656. doi: 10.1002/advs.202105656 (PMC8981444; doi:10.1002/advs.202105656)
Supplement: Supplementary file 1 — Supporting Information [file ADVS-9-2105656-s001.pdf]

ADVANCED SCIENCE
Open Access

# Supporting Information

for *Adv. Sci.*, DOI 10.1002/advs.202105656

Electronic Localization Derived Excellent Stability of Li Metal Anode with Ultrathin Alloy

*Danqi He, Wenjun Cui, Xiaobin Liao, Xianfei Xie, Mingheng Mao, Xiahan Sang, Pengcheng Zhai, Yan Zhao*, Yunhui Huang* and Wenyu Zhao**

ADVANCED SCIENCE
Open Access

# Supporting Information

for *Adv. Sci.,* DOI: 10.1002/advs.202105656

# Electronic localization derived excellent stability of Li metal anode with ultrathin alloy

*Danqi He, Wenjun Cui, Xiaobin Liao, Xianfei Xie, Mingheng Mao, Xiahan Sang, Pengcheng Zhai, Yan Zhao*, Yunhui Huang*, and Wenyu Zhao**

D. He, P. Zhai
Hubei Key Laboratory of Theory and Application of Advanced Materials Mechanics, Wuhan University of Technology, Wuhan 430070, China

W. Cui, X. Liao, M. Mao, X. Sang, Y. Zhao, W. Zhao
State Key Laboratory of Advanced Technology for Materials Synthesis and Processing, Wuhan University of Technology, Wuhan 430070, China
E-mail: yan2000@whut.edu.cn; wyzhao@whut.edu.cn;

X. Xie
State Key Laboratory of Advanced Electromagnetic Engineering and Technology, School of Electrical and Electronic Engineering, Huazhong University of Science and Technology, Wuhan 430074, China

Y. Huang
State Key Laboratory of Material Processing and Die and Mold Technology, School of Materials Science and Engineering, Huazhong University of Science and Technology, Wuhan 430074, China
E-mail: huangyh@hust.edu.cn;

**Table S1** The detailed values of surface work function (SWF) for different phase.

| | Li (001) | LiBi (111) | LiAl (111) | LiAu (011) |
|---|---|---|---|---|
| Average potential (eV) | 1.3 | 1.57 | 2.96 | 2.65 |
| Fermi level (eV) | -1.59 | -2.85 | -1.13 | -0.93 |
| SWF (eV) | 2.89 | 4.42 | 4.09 | 3.58 |

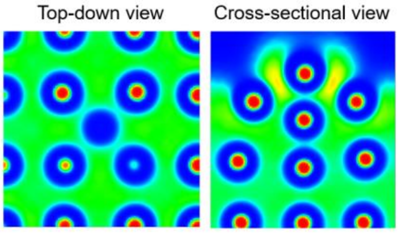


**Figure S1** ELF results of the Li(001) surface after platting $Li_6$ cluster.

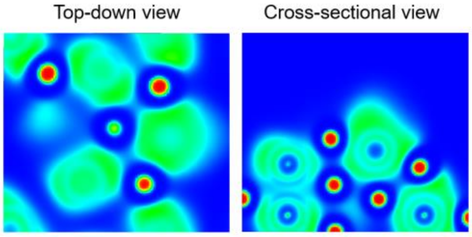


**Figure S2** ELF results of the LiBi(111) surface after platting $Li_6$ cluster.

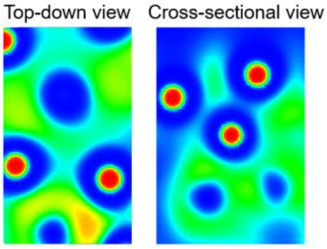


**Figure S3** ELF results of the LiAl(111) surface after platting $Li_6$ cluster.

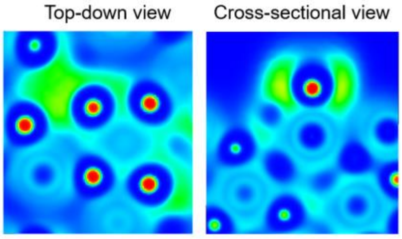


**Figure S4** ELF results of the LiAu(011) surface after platting $Li_6$ cluster.

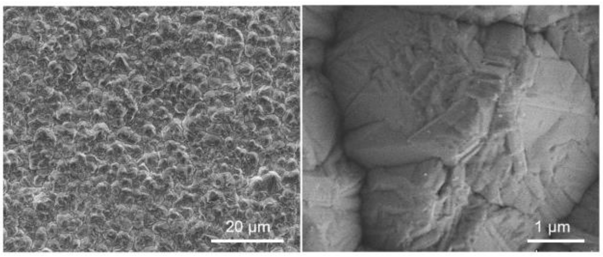


**Figure S5** The surface morphology of Cu foil.

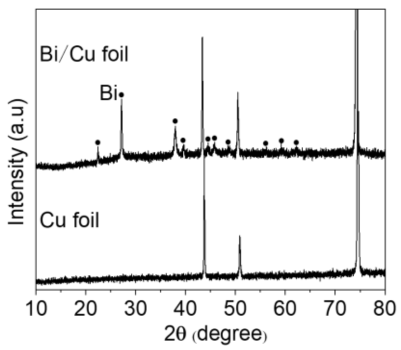


**Figure S6** XRD patterns of Cu foil and Bi/Cu foil.

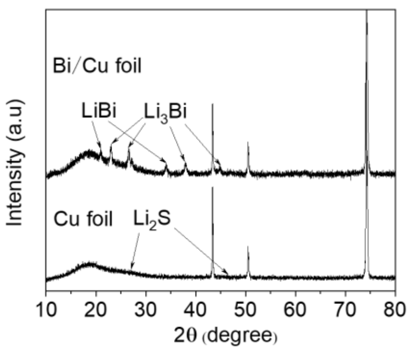


**Figure S7** XRD patterns of Cu foil and Bi/Cu foil after 2 depositing/stripping cycles.

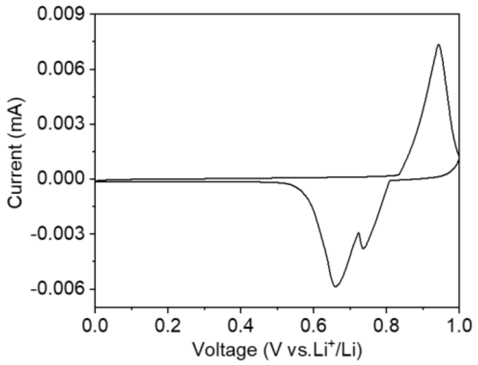


**Figure S8** CV curves at 0.1 mV $s^{-1}$ of the cell with Bi/Cu foil.

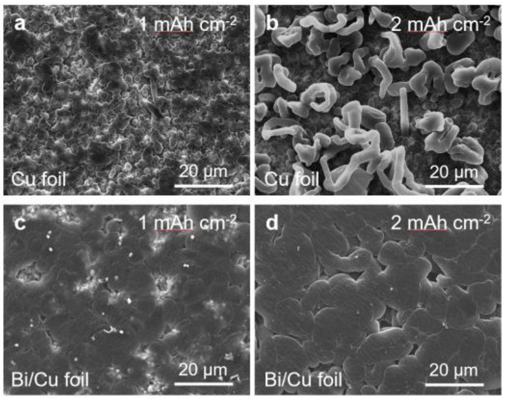


**Figure S9** SEM images of the morphologies of Li deposition on Cu foil and Bi/Cu foil at a current density of 1mA $cm^{-2}$ with different capacities.

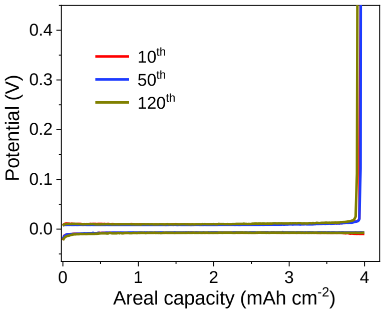


**Figure S10** Voltage profiles of Bi/Cu foil at a current density of 1 mA $cm^{-2}$ with total capacity of 4 mA h $cm^{-2}$ during different cycles.

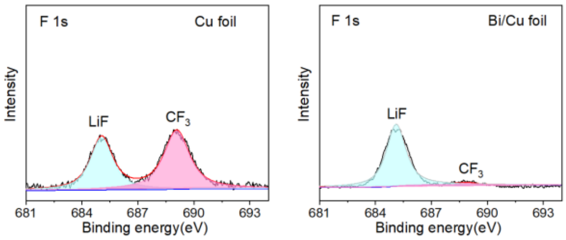


**Figure S11** XPS spectra of F 1s for different current collectors after cycling.

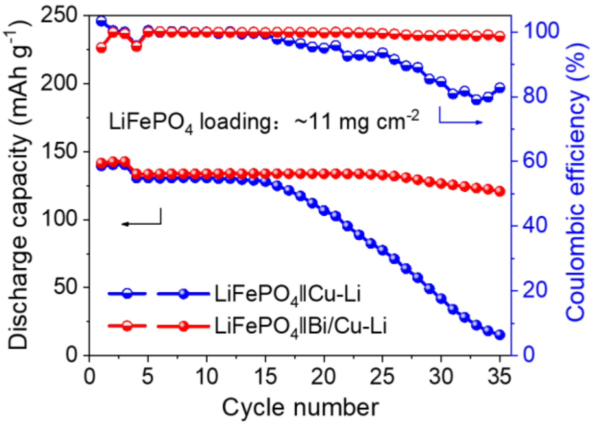


**Figure S12** Cycling performance of the full-cells consisting of a Cu-Li or Bi/Cu-Li anode and a high-loading LFP cathode.
